# Computational completion of the Aurora interaction region of N-Myc in the Aurora a kinase complex

**DOI:** 10.1038/s41598-023-45272-3

**Published:** 2023-10-26

**Authors:** Pinar Altiner, Süleyman Selim Çınaroğlu, Ahmet Can Timucin, Emel Timucin

**Affiliations:** 1 Institut de Pharmacologie et de Biologie Structurale (IPBS), CNRS, Université Toulouse III - Paul Sabatier (UT3), 31077 Toulouse, France; 2 Department of Biochemistry, University of Oxford, South Parks Road, Oxford, OX1 3QU UK; 3 Department of Molecular Biology and Genetics, Faculty of Engineering and Natural Sciences, Acibadem University, 34752 Istanbul, Turkey; 4 Department of Biostatistics and Medical Informatics, School of Medicine, Acibadem University, 34752 Istanbul, Turkey

**Keywords:** Computational biology and bioinformatics, Protein structure predictions, Oncogenes

## Abstract

Inhibiting protein–protein interactions of the Myc family is a viable pharmacological strategy for modulation of the levels of Myc oncoproteins in cancer. Aurora A kinase (AurA) and N-Myc interaction is one of the most attractive targets of this strategy because formation of this complex blocks proteasomal degradation of N-Myc in neuroblastoma. Two crystallization studies have captured this complex (PDB IDs: 5g1x, 7ztl), partially resolving the AurA interaction region (AIR) of N-Myc. Prompted by the missing N-Myc fragment in these crystal structures, we modeled the complete structure between AurA and N-Myc, and comprehensively analyzed how the incomplete and complete N-Myc behave in complex by molecular dynamics simulations. Molecular dynamics simulations of the incomplete PDB complex (5g1x) repeatedly showed partial dissociation of the short N-Myc fragment (61–89) from the kinase. The missing N-Myc (19–60) fragment was modeled utilizing the N-terminal lobe of AurA as the protein–protein interaction surface, wherein TPX2, a well-known partner of AurA, also binds. Binding free energy calculations along with flexibility analysis confirmed that the complete AIR of N-Myc stabilizes the complex, accentuating the N-terminal lobe of AurA as a binding site for the missing N-Myc fragment (19–60). We further generated additional models consisting of only the missing N-Myc (19–60), and the fused form of TPX2 (7–43) and N-Myc (61–89). These partners also formed more stable interactions with the N-terminal lobe of AurA than did the incomplete N-Myc fragment (61–89) in the 5g1x complex. Altogether, this study provides structural insights into the involvement of the N-terminus of the AIR of N-Myc and the N-terminal lobe of AurA in formation of a stable complex, reflecting its potential for effective targeting of N-Myc.

## Introduction

MYCN (neuroblastoma-myelocytomatosis) is a viral gene located in the short arm of chromosome 2 (2p24.3), encoding the N-Myc oncoprotein^1^. MYCN has a marginal expression in adult tissues that markedly decreases after embryogenesis^2–4^. Through genomic amplification, chromosomal translocation or mutagenesis in the signaling pathways, MYCN expression may abnormally rise leading to elevated N-Myc levels in human cancers particularly in neuroblastoma^5–10^. This paradigm has long rendered N-Myc as an attractive target for treatment of tumors that are characterized by its amplification/overexpression^11–13^.

Inhibiting protein–protein interactions of the Myc family that regulates their proteasomal degradation is a viable approach to attenuate elevated N-Myc levels^14^. Degradation of Myc proteins is regulated by two degrons (T58 and T244) that recruit an FBW7 dimer forming the SCF E3 ubiquitin ligase complex^15,16^. Because N-Myc lacks the second degron (T244), its ubiquitylation mainly relies on the first degron and its interaction with an FBW7 dimer^16,17^. The T58 degron of N-Myc is found at the binding interface of the complex formed by the Aurora kinase A (AurA) and N-Myc, thus the interaction of N-Myc with AurA inhibits its ubiquitinylation in neuroblastoma by blocking the interaction of N-Myc with the E3 ubiquitin ligase SCFFbxW7^17,18^. As a result, of many other intermolecular interactions, N-Myc’s interaction with AurA is highly relevant for pharmacological modulation of N-Myc^19^.

**Figure 1:**
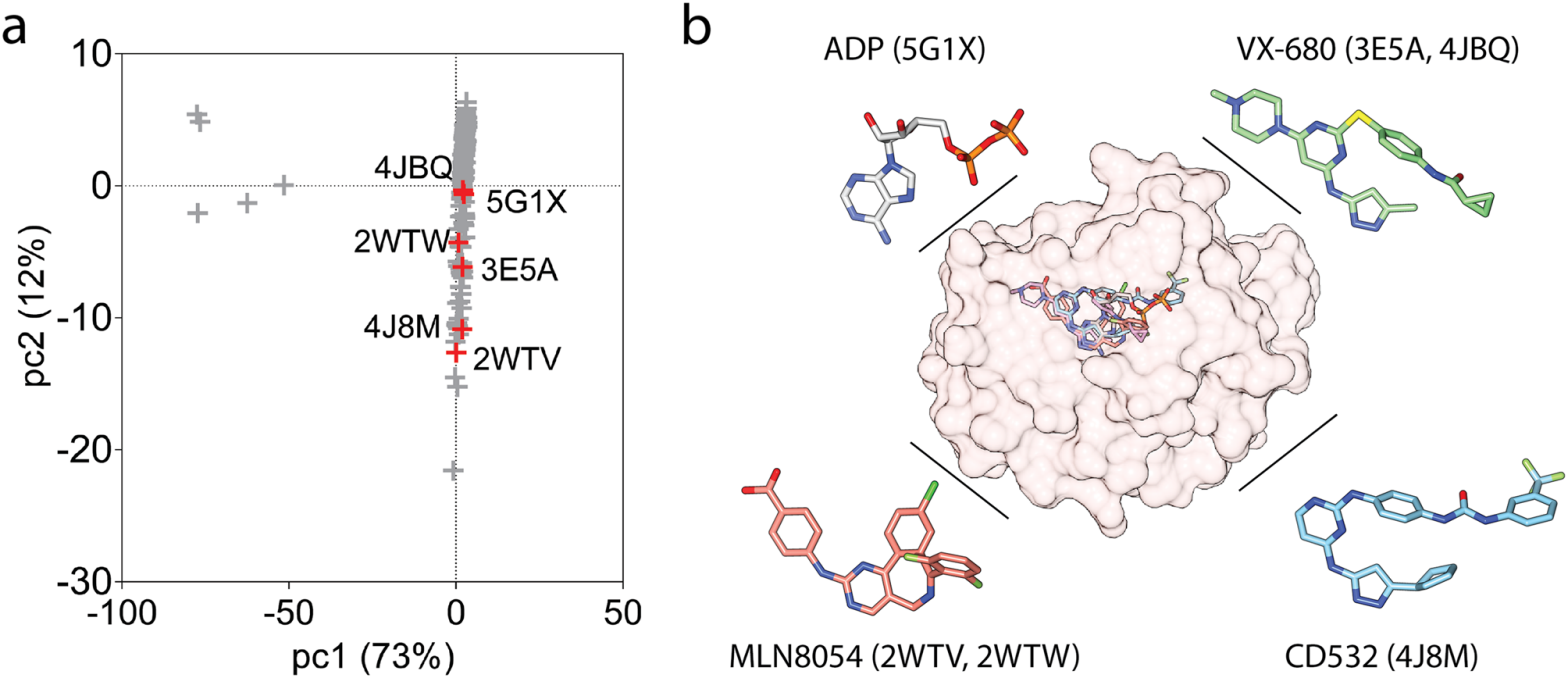
(**a**) Score plot of the first two principal components based on the change in the C$\alpha$ coordinates of the PDB ensemble. Selected PDBs were labeled. List of the all structures can be found in supplementary information (Supplementary Table S1). (**b**) Active site of AurA KD in the 5g1x complex. Superimposed poses of the ADP and KIs were shown (O: red, N: blue, S: yellow, P: orange, F/Cl: green). Structures were visualized by UCSF Chimera (1.17.3).

**Figure 2:**
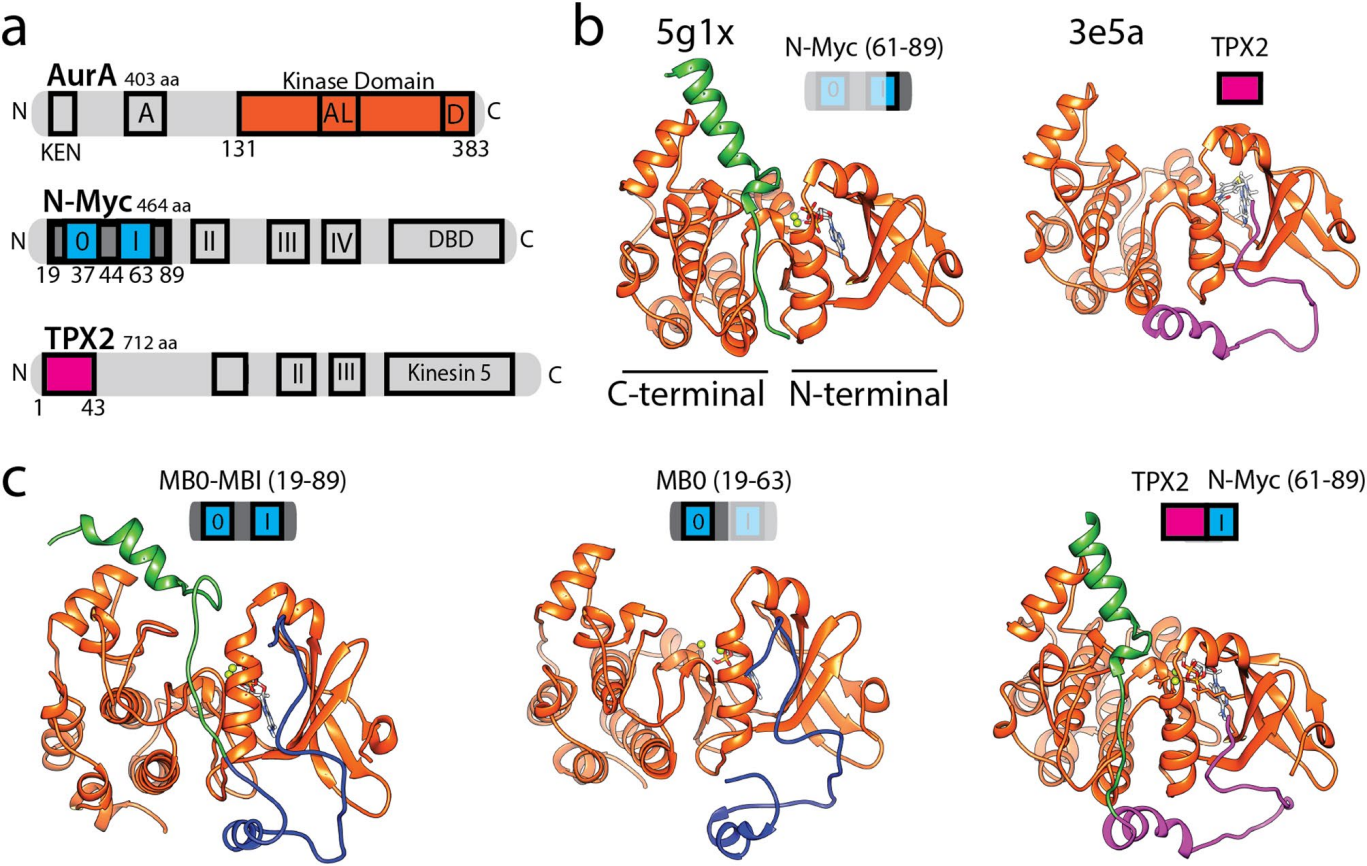
(**a**) Domain architecture of AurA, N-Myc and TPX2 were shown coloring the structures utilized in this study (AurA kinase domain: orange, N-Myc MB motifs: blue, TPX2 Aurora binding epitope: pink). (**b**) Crystal structures of AurA protein–protein complexes that were retrieved from the PDB and analyzed in this study were illustrated (AurA: orange, N-Myc missing motif: blue, N-Myc 61–89: green, TPX2: pink). (**c**) The modeled structures were shown and colored accordingly. Structures were visualized by VMD (v1.9.4a).

**Table 1:** Details of the AurA complexes analyzed in this study.

Source	Protein partner (residues)	Ligand	AurA details*
5G1X	N-Myc (61–89)	ADP	A/T288/C290A-C393A
Model	N-Myc MB0 (19–60)	ADP	A/T288/C290A-C393A
Model	N-Myc MB0-MBI (19–89)	ADP	A/T288/C290A-C393A
3E5A	TPX2 (7-43)	VX-680	A/T288/*NA*
Model	TPX2 (7-43), N-Myc (61–89)	ADP	A/T288/C290A-C393A
4JBQ	*NA*	VX-680	A/*NA*/T287D
4J8M	*NA*	CD532	I/*NA*/T287D
2WTW	*NA*	MLN8054	A/T288/L215R-T217E-R220K
2WTV	*NA*	MLN8054	I/T287-T288/L215R-T217E-R220K
2WTV	*NA*	MLN8054	I/*NA*/L215R-T217E-R220K

*NA* not available.

*AurA details: Activation loop, A: active, I: inactive/phosphorylation/mutation.

AurA is a member of the Aurora kinase family that plays a role in the regulation of the G2/M check-point of cell cycle^20^. Similar to N-Myc, AurA has also been shown to be amplified in human tumors^20^. Thus, several small molecule inhibitors that target the kinase function of AurA are available today^21^. One class of inhibitors was shown to destabilize the AurA–N-Myc complex structure^19^ and induce N-Myc degradation in vitro^22^. Nonetheless, clinical trials concluded a limited efficacy for these inhibitors in childhood neuroblastoma^22,23^, reiterating the need for direct inhibitors targeting the AurA–N-Myc interaction^24^.

Determination of how AurA interacts with the N-Myc Myc Box (MB) motifs is an integral part of the development of such direct inhibitors of the AurA–N-Myc complex^25,26^. The Myc family, including N-Myc, forms protein–protein interactions through their transactivation domains (TADs) that are composed of intrinsically disordered Myc Box (MB) motifs^4,27–29^. Although AurA–N-Myc complex has been associated with two PDB structures^16,19^, neither of the structures include the MB motifs. Thus, despite two experimental structures of AurA–N-Myc complex, the exact interaction network between AurA and N-Myc is not known in its entirety.

**Figure 3:**
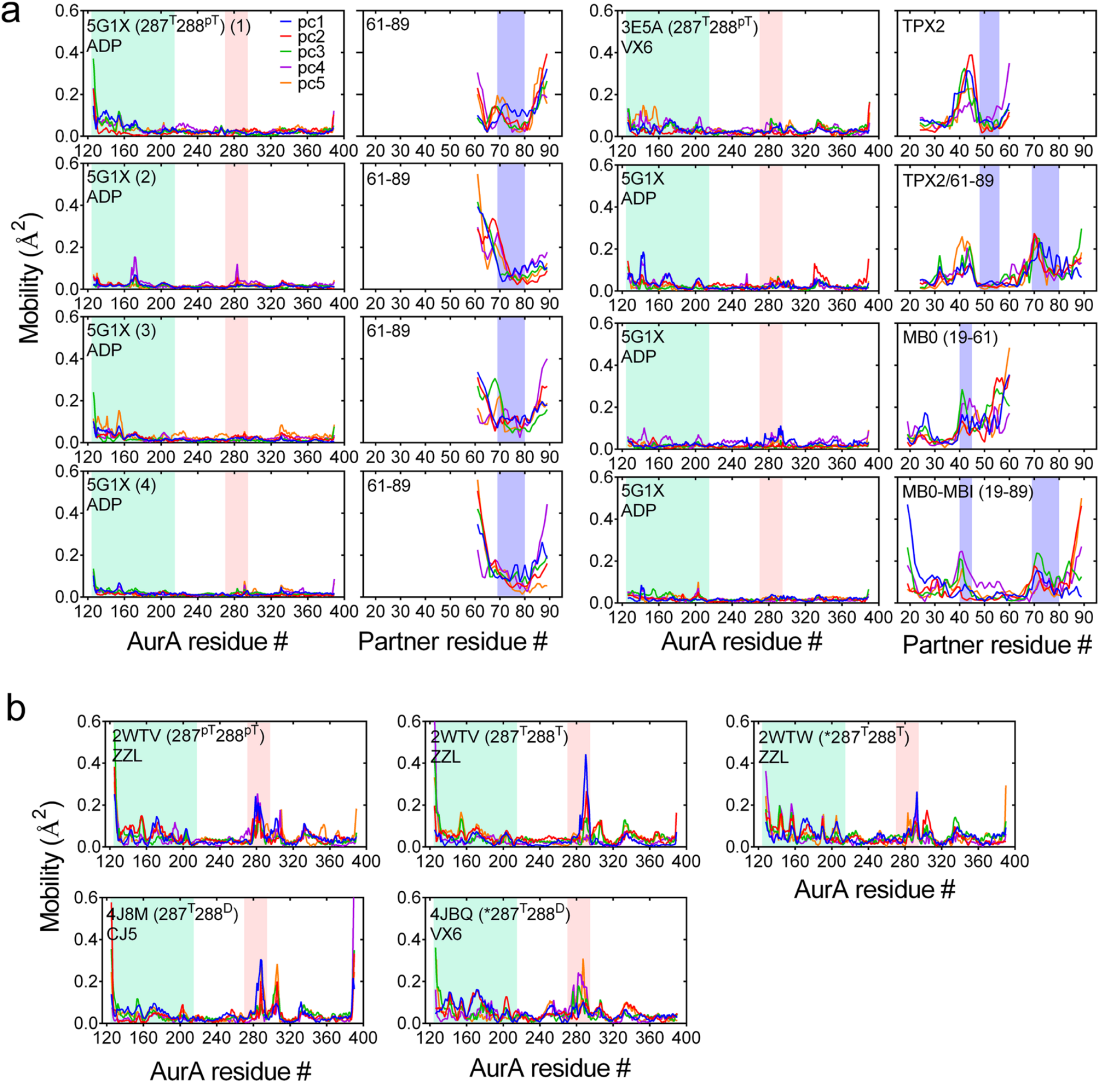
C$\alpha$ fluctuations of AurA (left) and protein partners (right) were calculated from pca using the residue loadings onto first five pcs. Details of pca were given in Supplementary Fig. S3. AurA N-terminal lobe and the activation loop that resides at the central region were shared green and pink, respectively. The helical regions that were spotted in the initial structures of the protein partners of AurA were shaded blue.

**Figure 4:**
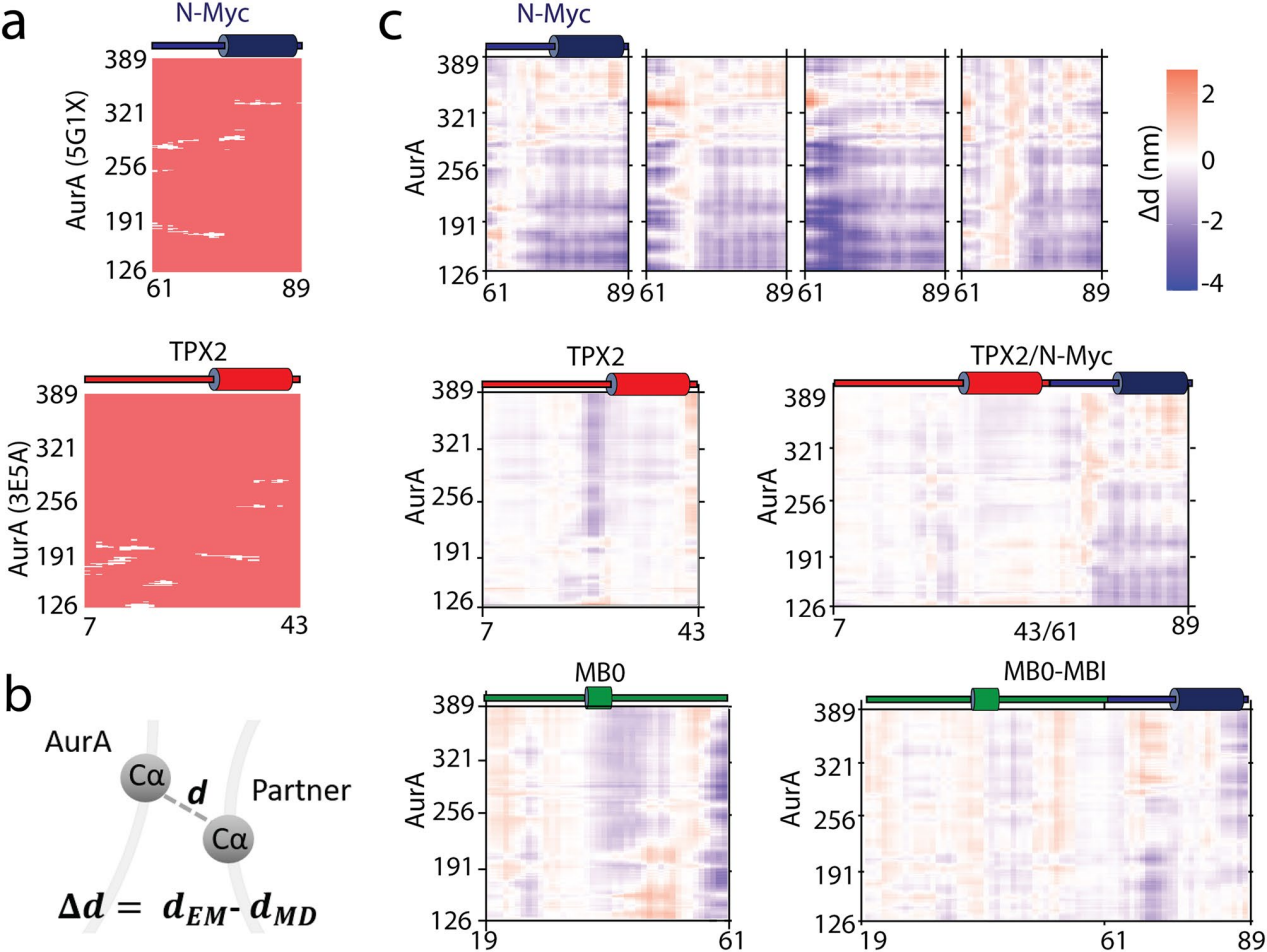
(**a**) Panel shows the contact map of the crystal structures of AurA–N-Myc (5g1x) and AurA–TPX2 (3e5a) wherein white marks are indicative of contacts closer than 5 Å. (**b**) A change in the distance of C$\alpha$s was calculated by subtracting d$_{MD}$ from d$_{EM}$, corresponding to a given C$\alpha$–C$\alpha$ distance at the start and end of the simulation, respectively. (**c**) Heatmaps show the change in the C$\alpha$ distances between AurA and its protein partners. A red color indicates approaching of two C$\alpha$s while a blue color indicates their separation.

**Table 2:** MM-GBSA based binding free energy predictions.

Partner	Residues (atoms)	Static models*	MD poses**	LE***
61–89 (1)	29 (232)	− 93.09	− 48.06 ± 24.29	− 0.21
61–89 (2)	29 (232)	− 63.17	− 53.22 ± 21.72	− 0.23
61–89 (3)	29 (232)	− 36.58	− 42.12 ± 8.66	− 0.18
61–89 (4)	29 (232)	− 57.27	− 48.50 ± 25.03	− 0.21
61–89 (5)	29 (232)	− 83.55	− 55.71 ± 20.78	− 0.24
MB0-MBI	71 (576)	− 137.57	− 159.34 ± 22.71	− 0.28
TPX2	37 (300)	− 117.91	− 130.94 ± 22.26	− 0.44
MB0	42 (345)	− 81.14	− 100.90 ± 17.62	− 0.29
TPX2/N-Myc	66 (532)	− 173.98	− 164.97 ± 12.96	− 0.31

*$\Delta \Delta G$ (kJ mol$^{-1}$) for the starting structures.

**$\Delta \Delta G$ for five MD poses from the last half of the simulations given as “mean±SD”.

***Ligand efficiency (LE): $\Delta \Delta G$ per heavy atom.

In this study, we focused on the PDB structure of AurA–N-Myc (5g1x), and extensively analyzed its structure and dynamics using molecular dynamics simulations. Relying on this PDB complex, we modeled the missing regions of N-Myc that includes the MB motifs and the missing T54 degron. Our results demonstrated that the completion of the N-Myc structure in the unstable 5g1x complex led to its stabilization unveiling a novel interaction surface between AurA and N-Myc for drug targeting studies. Our results provides structural insights into the role of MB motifs of N-Myc and the N-terminal lobe of AurA for formation of a stable complex elucidating the complete AurA and N-Myc complex. Ultimately, this work reflects the complementarity of computational methods to the experimental ones for identification of molecular interactions.

**Figure 5:**
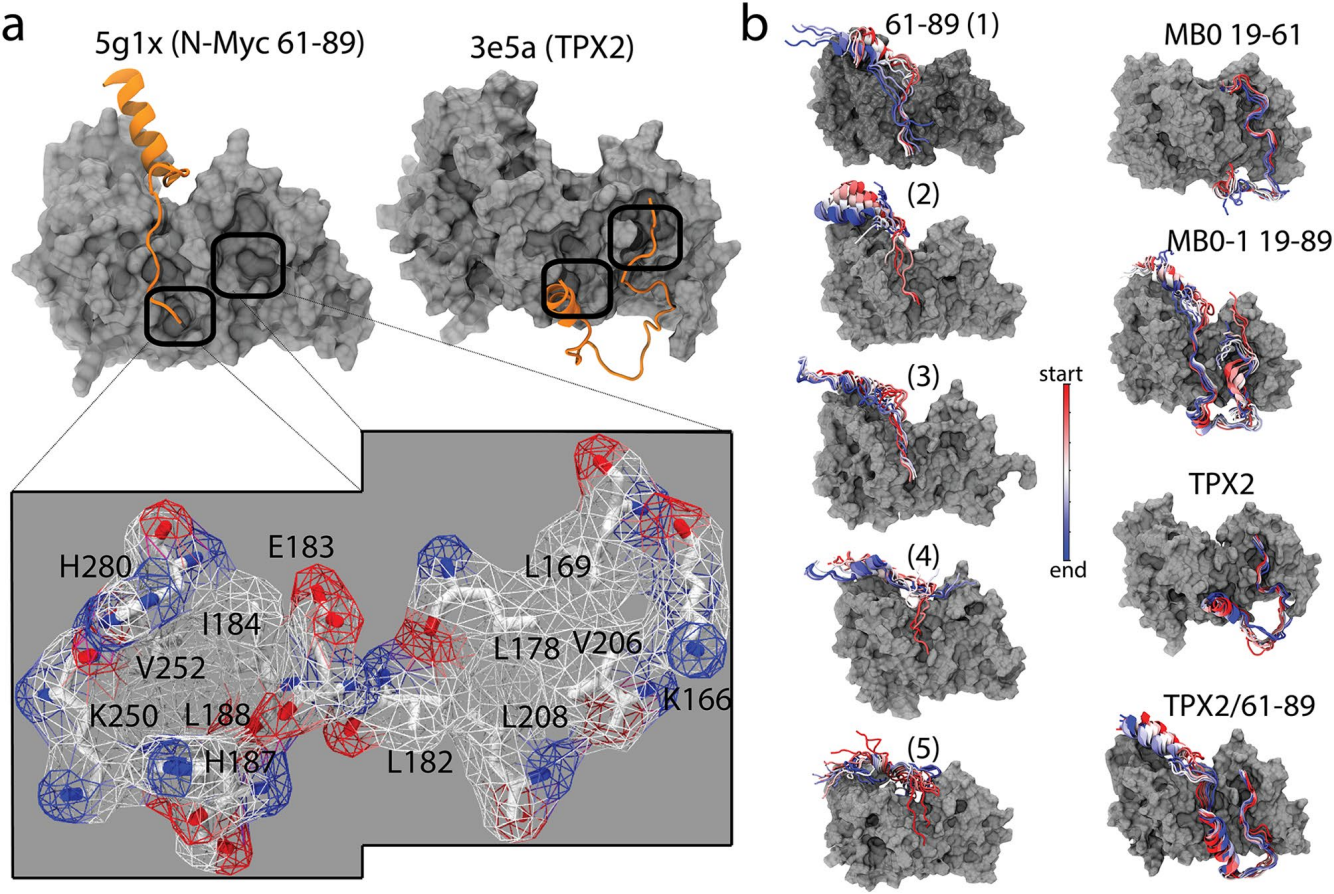
(**a**) Panel shows the tunnels spotted in the crystal structures of AurA–N-Myc and AurA–TPX2 protein–protein complexes. Close-up view focuses on the amino acids forming the identified tunnels, details of which are given in Table 3. (**b**) Panel shows the reduced trajectories of the protein partners on AurA throughout the 500 $\upmu$s. For the 61–89 fragment numbered (5), the simulation duration was 1 $\upmu$s. Reduced trajectories were visualized by VMD (v1.9.4a).

**Table 3:** Details of AurA tunnels.

No.	Composition	Radius	Length	Curvature
1	E183 I184 H187 L188 K250 V252 H280	2.1	1.4	1.3
2	K166 L169 L178 V182 V206 L208	1.5	1.5	1.0

Radius, length, curvature are in Å.

Both tunnels had a throughput score higher than 0.90.

## Results

### Selection of Aurora kinase A structures

To assess the structure and dynamics of AurA–N-Myc complex in comparison to other protein–protein complexes of AurA, we made a thorough PDB search, collecting 159 AurA kinase domain (KD) structures (Supplementary Table S1). Their structural similarity was evaluated by a principal component analysis (pca) on the C$\alpha$ coordinates, excluding the activation loop, which was often missing in the crystal structure of AurA. Score plot of pc1–pc2 is shown in Fig. 1a. Because the first pc explained more than 70% of the variation in the C$\alpha$ positions of this PDB ensemble, similarity was assessed based on the pc1 scores (Fig. 1a). To ensure a similar backbone to the 5g1x complex in the selected complexes, we focused on the AurA structures close to the 5g1x complex. Based on this rationale, we selected the PDB structure of 3e5a, another protein–protein complex of AurA formed with TPX2. These two protein–protein complexes (5g1x and 3e5a) were used as the reference structures during modeling of the complete N-Myc.

**Figure 6:**
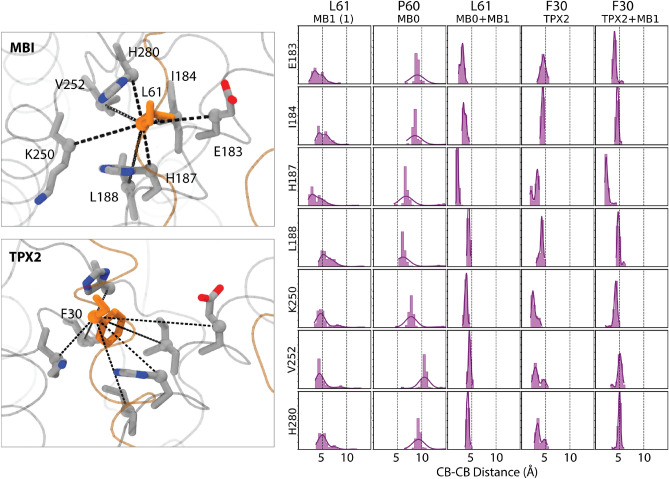
Distribution of C$\beta$–C$\beta$ distances between the first tunnel amino acids and the closest amino acid in the protein partners. For the 5g1x complex, the first simulation was shown. Rest of the analysis can be found in the Supplementary Fig. S6. Left-panel illustrates how the tunnel amino acids of AurA and partners of N-Myc and TPX2 structures are placed. C$\beta$–C$\beta$ distances that were monitored were marked by dashed lines. 5g1x and 3e5a crystal structures were used for visualization by VMD (v1.9.4a).

**Figure 7:**
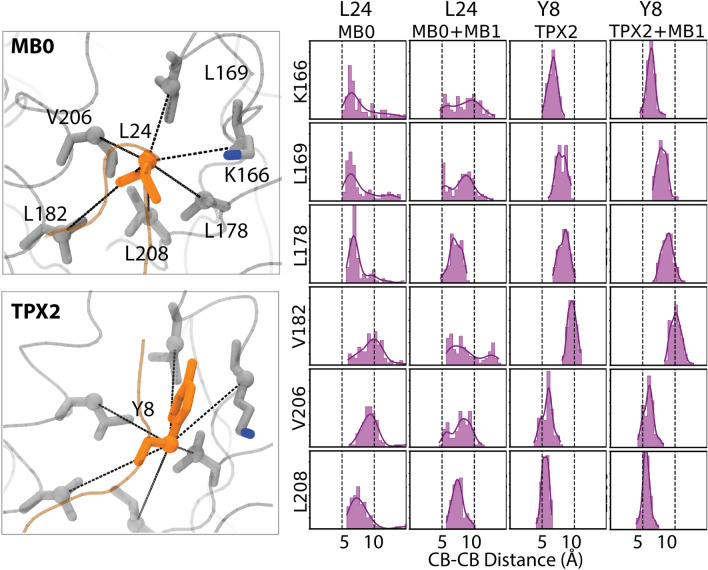
Distribution of C$\beta$–C$\beta$ distances between the second tunnel of AurA and the closest amino acids in the protein partners; for N-Myc MB0 the closest residue is L24 while for TPX2 it is Y8. The N-Myc model (19–89) and 3e5a crystal structures were used for visualization by VMD (v1.9.4a).

**Figure 8:**
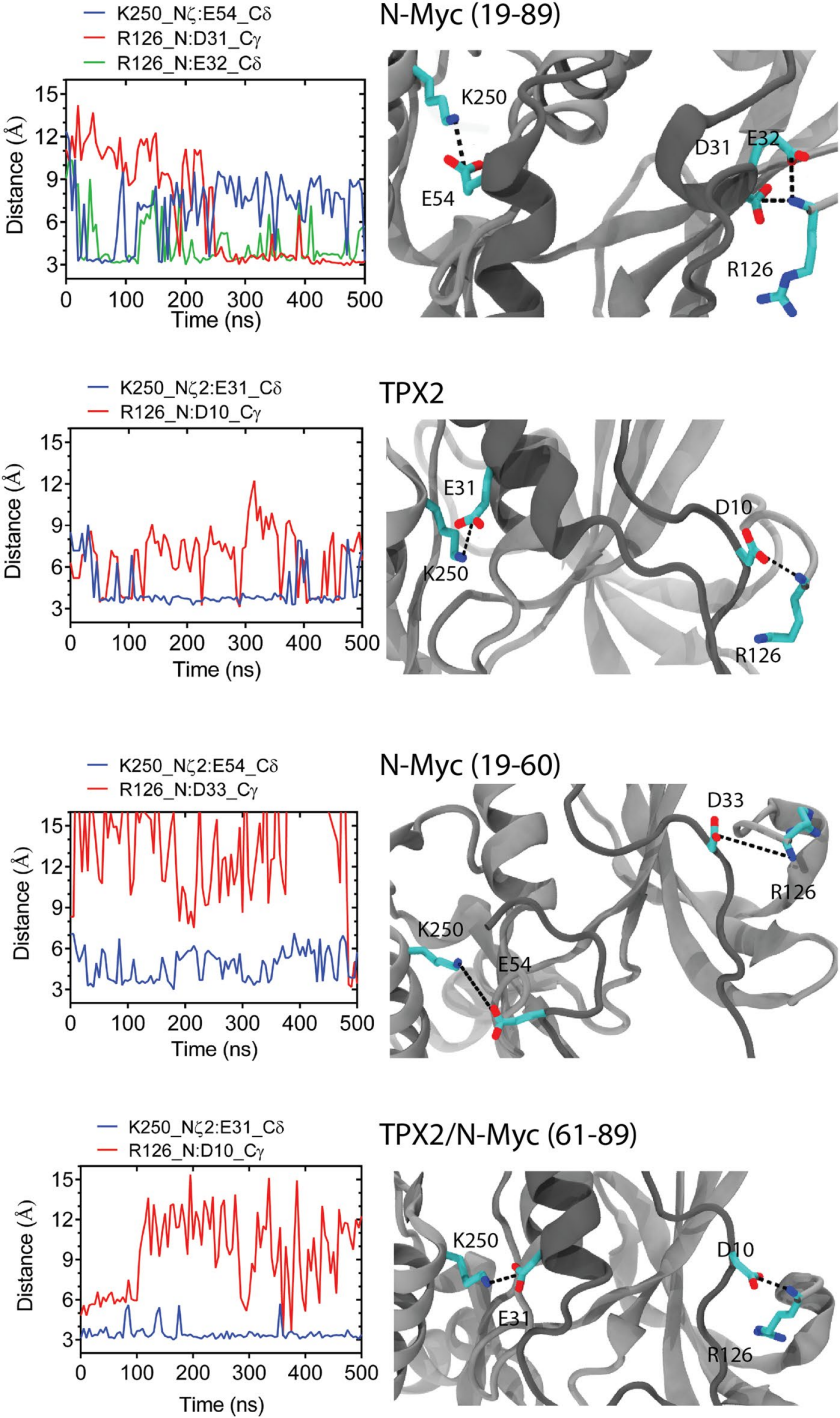
Electrostatic interactions between AurA and partners. Left panels show the distance between the identified electrostatic interactions. Right panels show close-up views of these interactions by VMD (v1.9.4).

The active site of AurA protein–protein complexes including N-Myc and TPX2 complexes were often accommodated by either an ADP molecule or a kinase inhibitor (KI) (Fig. 1b). To distinguish the impact of AurA–protein interactions from AurA–KI interactions on the complex dynamics, additional AurA structures that were only complexed with a KI were included in the study. Among these structures, 4bjq shares the same KI with the 3e5a structure of the AurA–TPX2 complex (Fig. 1b). The 4j8m structure, which has an altered activation loop conformation that was proposed to affect N-Myc binding^19,30^, was also recruited to the study. Because the conformation and phosphorylation state of the activation loop of AurA KD play important roles in AurA activation^17,31^ and formation of the N-Myc complex^19^, we lastly selected two structures with alternative loop conformations and phosphorylation states (PDB IDs: 2wtv and 2wtw). Overall, six AurA PDB complexes with similar backbone coordinates except the activation loop were recruited to this study, allowing a comprehensive analysis of the AurA and N-Myc complex in comparison to different partners of TPX2 and/or KIs.

### Completing the Aurora kinase A and N-Myc complex structure

Figure 2a shows the domain architectures of AurA and its protein partners of N-Myc and TPX2. The latter is a microtubule-associated protein that localizes AurA to microtubules for spindle assembly^32^. Kinase domain of AurA and TPX2 forms a well-characterized complex with AurA in over 25 PDB structures. TPX2 has a short AurA binding epitope in its N-terminus (Fig. 2a), which interacts with the N-terminal lobe of the kinase^32^ (Fig. 2b). Compared with the AurA–TPX2, the AurA–N-Myc complex is a less studied complex, which was represented by two PDB structures (PDB IDs: 5g1x, 7ztl)^16,19^. The N-Myc fragments in both structures were not complete to address the entire interaction interface^19^. The AurA interaction region (AIR) comprised both MB motifs corresponding to the residues between 28–89 (Fig. 2a). Specifically, the 5g1x complex contains the N-Myc fragment corresponding to the residues 61–89 and the 7ztl complex contains a shorter fragment corresponding to the residues 72–89. The MB0 motif (19–47) was missing in both of the structures (Fig. 2b) while only a few amino acids from MBI motif (61–63) were captured in the 5g1x complex. In summary, neither of the PDB structures captured neither of the full MB motifs of N-Myc (Fig. 2b).

To determine the complete interface between AurA and N-Myc, we have modeled the missing AIR in the 5g1x structure, relying on both experimental findings and known protein–protein interactions of AurA. The captured N-Myc fragment (61–89) in both PDB structures positioned at the central domain of AurA, having their N-termini oriented towards the lower part of the central AurA region (Fig. 2b). The N-terminal lobe of AurA has been recognized to involve in many protein–protein interactions with distinct partners such as TPX2 and TACC3^33^. More importantly, binding of the missing AIR motif was shown to compete with the binding of TPX2^19^, reflecting an overlap in their binding sites. Leveraging this information, we have modeled the missing AIR of N-Myc in the 5g1x complex on the TPX2 binding site, which is at the N-terminal lobe of AurA. Initially, we copied the coordinates of the TPX2 fragment from the 3e5a structure to the 5g1x model and mutated to generate the missing N-Myc sequence. Lastly, we extended the N-terminus of the N-Myc model to cover the entire MB0-I motifs corresponding to 19–89. Resulting structure representing the complete AIR of N-Myc in complex with the AurA was illustrated in Fig. 2c. Similarly relying on the TPX2 backbone coordinates, we also generated two additional N-Myc containing AurA complexes that harbor only the missing N-Myc fragment (19–60) and a chimeric TPX2 and N-Myc fragment (Table 1 and Fig. 2c). Taken together with the completed AurA–N-Myc complex, these additional complexes allowed us to scrutinize the individual and synergistic contributions of both AurA interaction regions of N-Myc and TPX2 to complex stability and dynamics (Fig. 2c and Table 1).

### Investigating the backbone structure and dynamics of the AurA complexes

The modeled structures along with the crystal complexes were analyzed by molecular dynamics (MD) simulations (Table 1). Supplementary Fig. S2 shows the RMSD heatmaps computed for the displacements of C$\alpha$ atoms. The crystal complex of 5g1x was the most mobile complex. Four replicate simulations of this system repeatedly showed a highly dynamic complex backbone. Unlike the 5g1x complex, the rest of the AurA complexes including the computed models did not show a high level of mobility. TPX2 and the completed MB0-MBI motifs of N-Myc displayed highly stable backbones. Overall, RMSD analysis suggested that the missing MB0 motif of N-Myc greatly affects dynamics of the complex as its inclusion in the 5g1x structure stabilized the complex backbone. The AurA–KI structures showed almost a frozen AurA backbone with the exception of the 4bjq structure, which was only different from the 3e5a structure by the absence of the TPX2 fragment. Hence, comparison of 4jbq and 3e5a structures unraveled the stabilization impact of the TPX2 on AurA^32^.

We extracted the essential dynamics of the complexes from the last half of the trajectory (Figs. 3a and S3) and analyzed the C$\alpha$ fluctuations using the entire trajectory (Supplementary Fig. S4). We observed a slight increase in the flexibility of the N-terminal lobe of AurA for the 5g1x complexes compared to the other systems. The 61–89 fragment of N-Myc consistently showed high flexibility, accentuating that high mobility observed for the 5g1x complex (Supplementary Fig. S2) was due to N-Myc rather than AurA. Especially, the termini of the 61–89 region of N-Myc was highly mobile as reflected by the U-shaped fluctuations for the 5g1x complex (Fig. 3a), indicating that this region could not maintain a stable conformation.

All protein partners of AurA showed mobile C-termini (Figs. 3a-blue shade and S4), implying a loose interaction through their C-termini. Particularly, this observation underscored that the long constructs including the full AIR of N-Myc and the fused TPX2/N-Myc (61–89) along with the N-Myc fragment (61–89) in the 5g1x complex loosely interacted with the upper region of the central-lobe of AurA (Fig. 2). On the other hand, for the TPX2 and MB0 motifs, a loose C-termini would mean a loose interaction with the lower region of the central-lobe of AurA. These results overall indicated loose interactions of the partners of either TPX2 or N-Myc origin with the lower and upper parts of the central lobe of AurA.

The modeled MB0 motif and the full AIR of N-Myc were almost frozen for the region between 19–40, i.e. flat fluctuations (Figs. 3a-blue shade and S4). The TPX2 and the fused TPX2/N-Myc fragments also showed minimal fluctuations for the 40–50 region (Fig. 3a-blue shade), reflecting tight interactions of the MB0 and TPX2 containing fragments with the kinase. Particularly, the longer partners of either the complete AIR of N-Myc or the TPX2/N-Myc (61–89) strongly interacted with AurA. These observations are indicative of stable complexes for the partners interacting with the N-terminal lobe of AurA, reflecting the significance of the N-terminal lobe of AurA for dimer stability rather than the central region, wherein the activation loop resides.

The phosphorylated T287 and T288 positions in the 2wtv structure were de-phosphorylated to investigate the impact of phosphorylation. The activation loop was more flexible in the non-phosphorylated form than it was in the phosphorylated form (Fig. 3b). Other than these regions, AurA–KI complexes showed no significant backbone mobility in response to changes in the conformation and/or phosphorylation state of the activation loop (Fig. 3b).

### Separation of AurA backbone from the protein partners

Figure 4a depicts the contact maps of the PDB structures of 5g1x and 3e5a. TPX2 mainly interacted with the N-terminal lobe of the kinase while the 61–89 fragment of N-Myc interacted with the central region of AurA. We measured the change in the distance between C$\alpha$ atoms of AurA and its protein partners after simulations (Fig. 4b). This analysis utilized only two snapshots from the production simulations, i.e. the first- and last-frame snapshots, otherwise discarding the rest of the trajectory. Although focusing on only two snapshots would be misleading, we measured the relative change in the backbone separation of AurA and its partner using the first- and last-frame snapshots for a quantitative description of the movement of the partners on the AurA surface, i.e. whether they maintained or changed their initial poses.

The backbone of the N-Myc fragment in the 5g1x complex (61–89) displayed a large separation from the AurA backbone, mirroring a large conformational change in the N-Myc. The C-termini of the MB0 and TPX2/61–89 N-Myc partners were also slightly displaced in line with their fluctuation analysis (Fig. 3). However, other protein partners stably maintained their initial positions on AurA for 500 ns. This analysis showed separation of the N-Myc fragment (61–89) from the AurA in the 5g1x complex; otherwise other protein partners did not undergo an apparent separation from AurA.

We estimated the binding free energy of the AurA–protein complexes by the MM-GBSA method using five MD snapshots from the last half of the simulations (Table 2). Predictions showed that the 61–89 fragment of N-Myc was the weakest binder among other partners. Repeated simulations of this fragment suggested that it binds to AurA with an estimated average of − 50 kJ mol$^{-1}$. However, the longer constructs (MB0-I and fused) or the fragments that only bind to the N-terminal lobe of AurA (MB0 and TPX2) showed much higher lower values ranging in from − 164 to − 100 kJ mol$^{-1}$. We analyzed the binding free energies of the starting structures which consist of two crystal structures (3e5a and 5g1x) and three models. Parallel with the binding free energy estimates from the MD poses, the starting structures of the constructs that bind to the N-terminal lobe of AurA formed more stable complexes than those formed by the N-Myc 61–89 fragment. Because the partner sizes differ, the predicted scores were normalized to the number of heavy atoms, an analysis which showed that the TPX2 is the most effective binder closely followed by the MB0 containing N-Myc fragments. Another calculation that was made using ten different MD poses produced similar results (Supplementary Fig. S5).

### Important AurA surfaces for protein–protein interactions

We have identified two tunnels on the AurA surface that overlaps with the binding interfaces of the PDB complexes (5g1x, 3e5a) (Fig. 5). The first tunnel that was constructed from a pocket of 637 Å$^3$ is located at the lower part of the central AurA region. The second tunnel that came from a larger pocket of 974 Å$^3$ is found at the N-terminal lobe of AurA (Fig. 5a and Table 3). Both tunnels were similar in length and composition; however, the second tunnel on the N-terminal lobe is smaller in radius and curvature than is the first tunnel, meaning that the second tunnel forms a more confined space than the first tunnel (Table 3).

Figure 5a shows that two tunnels are lined up side-by-side and formed by hydrophobic amino acids with aliphatic sidechains and charged amino acids, whose charged groups point to the tunnel entrance while their sidechain hydrocarbons, together with other hydrophobic amino acids, form a hydrophobic pit (Fig. 5). Inspection of both crystal structures showed that both the 61–89 fragment of N-Myc and TPX2 could extend over the first tunnel while only TPX2 could reach over the second tunnel at the N-terminus of AurA.

We visualized the protein partners on the AurA surface to assess the change in their positions relative to the tunnels (Fig. 5b). The 61–89 fragment of N-Myc, which could only interact with the first tunnel, moved away from the first tunnel. However, other partners did not move, maintaining their initial pose with respect to the tunnels. This visual observation, which was in line with the C$\alpha$ separation analysis (Fig. 4), suggested the involvement of AurA surface tunnels in protein–protein interactions (Fig. 5b).

### Interactions of N-Myc and TPX2 with the AurA

The 61–89 fragment of N-Myc has an aliphatic leucine (L61) at the position that closely interacted with the first tunnel while TPX2 has an aromatic phenylalanine (F30) at the corresponding position (Fig. 6). For the MB0 motif (19–60), the closest position to the first tunnel is the P60. We monitored the distance between the tunnel residues and the closest position of the protein partners (Fig. 6). Distribution of the C$\beta$–C$\beta$ distances confirmed that L61 of the completed AIR of N-Myc tightly interacted with the amino acids forming the first tunnel, whilst L61 in the 61–89 fragment was separated from the first tunnel (Figs. 6 and S6). Expectedly, P60, whose initial position was not close to the tunnel, could not form stable interactions with the tunnel amino acids. F30 in the TPX2 closely interacted with the first tunnel amino acids in both TPX2-containing fragments.

N-Myc MB0 has an L24 while TPX2 has Y8 for the interaction with the second tunnel cluster. Distance distributions suggested that Y8 of TPX2 formed tighter interactions with the second tunnel than those formed by the L24 of MB0 (Fig. 7). While the L24 of MB0 interacted more closely with L208 and L178 in the tunnel, its interaction with other tunnel amino acids was less persistent as their distance distributions were wider, spreading to values as high as 10 Å(Fig. 7). On the other hand, Y8 of TPX2 closely interacted with all tunnel amino acids. TPX2 might have an advantage over N-Myc, because it carries two aromatic amino acids that strongly interact with the AurA tunnels compared to the N-Myc, which has aliphatic amino acids at the complementary positions. The assertion is particularly prominent for the second tunnel that forms a tighter space than the first tunnel (Table 3) and thus has a higher potential in hosting hydrophobic interactions^34^. In line with this, we surmise that interactions with the second tunnel alone stabilize the protein–protein interactions, as exemplified by the partners composed of only TPX2 and only MB0 motif (Fig. 7). However, the first tunnel alone is not sufficient to stabilize protein–protein interactions, as reflected by the partial dissociation of the 61–89 fragment of N-Myc in the 5g1x complex (Fig. 7). Altogether, we reported that protein partners of AurA extending to its N-terminal lobe and interact with the second tunnel form more stable interactions than those docked into the central lobe of AurA.

We also examined electrostatic intermolecular interactions between AurA and the partners (Fig. 8). The 61–89 fragment of N-Myc in the 5g1x complex did not form any persistent electrostatic interactions with the central domain of the AurA in any of the repeated simulations. For the other partners that are either longer than N-Myc (61–89) and/or extended towards the N-terminal lobe of AurA, we spotted two conserved interactions, wherein two basic amino acids of AurA, R126 and K250 interacted with acidic amino acids from the partner (Fig. 8). K250 is one of the amino acids forming the first tunnel (Fig. 5a) and R126 the first amino acid of AurA kinase domain, corresponding to the N-termini of the kinase structure (Fig. 2b). The acidic amino acids in the N-Myc (19–89) and TPX2 form persistent electrostatic interactions with K250 and R126. A glutamate, either E54 of the N-Myc (19–89) or E31 of the TPX2 interacted with the K250, whilst an aspartate, either D31 of N-Myc or D10 of TPX2 interacted with the free amino group of R126. Particularly, for N-Myc, the short stretch of acidic amino acids ($^{31}$DEDD$^{34}$) extending over the N-terminal lobe of AurA, forms an attractive site for this basic AurA surface. In the N-Myc (19–89), both D31 and E32 interacted with R126, while for the N-Myc (60–89) D33 interacted with the free amino group. Overall, these analyses have underscored that the missing N-Myc fragment and TPX2 form similar electrostatic and hydrophobic interactions with the N-terminal lobe of AurA, reflecting a common stabilization mechanism for both partners.

## Discussion

AurA and N-Myc interaction carries paramount importance for targeting elevated N-Myc levels in neuroblastoma cells^22,35^. Hitherto, two crystallization studies have focused on this interaction characterizing how the AurA interaction region (AIR) of N-Myc interacts with AurA^16,19^. Nonetheless, neither of the studies resolved the complete N-Myc fragment in this interaction, an outcome which is anticipated due to high disorder in the AIR of N-Myc^4,27^. Motivated by the missing N-Myc fragment in the AurA–N-Myc PDB complexes, we modeled the first complete structure between AurA and N-Myc, and analyzed how the complete model behaves in comparison to experimental findings and to other AurA complexes by molecular dynamics simulations.

The first crystal structure of AurA and N-Myc included the partial AIR fragment corresponding to the residues 61–89 (PDB ID: 5g1x)^19^. Although this partial N-Myc fragment was shown to independently bind to AurA, it does not entirely cover the AIR of N-Myc, which consists of both of the conserved MB motifs. Among these, MB0 could independently bind to AurA while MBI could not^19^. Despite the lack of a structural model between AurA and MB0 interaction, it was previously established that the binding site of MB0 overlaps with that of TPX2 on AurA^19^. In line with this, our modeling study ensured that the MB0 interacts with AurA through its N-terminal lobe, wherein TPX2 also binds^36^. Importantly, the simulations of the MB0 containing peptides captured stable complexes that possess an additional novel binding interface. Overall, the MB0 motif containing AurA structures identified in this study addressed the missing interaction between AurA and the AIR of N-Myc, complementing the empirical data. The active site of the kinase resides in the N-terminal lobe, with which TPX2 interacts and activates AurA^32^. Hence, we consider that the identified interaction of the MB0 of N-Myc and AurA, which also uses the N-terminal lobe of AurA, may provide a structural basis for the N-Myc-induced activation of AurA^19^.

The C-terminal part of the N-Myc AIR, which was captured in the crystal structure of 5g1x, was shown to independently bind to AurA^19^. However, we documented through molecular dynamics simulations that the same fragment did not form a stable complex with AurA; rather it, particularly its N-terminus, became separated from AurA. Our simulations consistently produced this result, contradicting with the experimental finding that showed that the N-Myc fragment of 61–89 could form stable interactions with AurA^19^. Recognizing that several factors in molecular dynamics simulations could impact outcome^37^, whether or not our simulations were carried out optimally is critical. From this perspective, we noted that a multi-step equilibration procedure was applied to all systems ensuring an optimal relaxation^38^. Second, the simulations of 5g1x complex were repeated for five times using different MD systems with varying box sizes (Supplementary Table S1). Additionally, we extended one of the 5g1x simulations to 1 $\upmu$s to further increase sampling. Overall, we have undertaken that the instability of the 5g1x complex was neither an apparent artefact due to a sub-optimal equilibration process nor occurred by chance. In line with these claims, a recent study also carried out molecular dynamics simulations of the same 5g1x complex and similarly found that the complex did not maintain the crystal pose^16^. In fact, the same study formed a stable interaction between the central region of AurA and a shorter N-Myc fragment (72-89) through cross-linking. Lastly, the C-terminal part of the N-Myc AIR in the crystal structure of 5g1x actively interacted with another AurA–N-Myc dimer in the unit-cell, suggesting stabilization of the incomplete N-Myc fragment in the 5g1x complex by crystal packing interactions. Overall, MD simulations of this complex collectively indicated that the N-Myc fragment between residues 61–89 did not form a stable complex with AurA, at least not through the binding interface identified in the crystal structure (Supplementary Fig. S7a).

Covalent peptides or small molecules could effectively target protein–protein interactions^39^, albeit the interaction surface to be targeted is pivotal to the outcome of such studies. Based on our analysis, all partners of AurA including TPX2 were loosely bound to the central domain of AurA (Figs. 3 and S4), wherein the aforementioned crystallization study targeted the N-terminal lobe of AurA by a cross-linked N-Myc fragment (72–89)^16^. Notwithstanding the promise of such covalent inhibitors against the AurA–N-Myc complex, we stress that the N-Myc interaction at the N-terminal lobe of AurA potentiates complex stability more than those at the C-terminal or the central lobe of AurA. Thus, the N-terminal lobe of AurA would be a better choice for, covalent or non-covalent, inhibition of the AurA–N-Myc complex, instead of the central region of AurA, with which N-Myc loosely interacted (Figs. 2b, S7b and Supplementary Video S1).

Two tunnels identified on AurA surface overlapped with the binding sites of both N-Myc and TPX2 (Fig. 5a). Although the conserved aromatic amino acids in the MB0 motif of N-Myc such F28, Y29, F35 and/or Y36 were proposed to complement the Y30 of TPX2^19^, which also interacted with the second tunnel, our models of N-Myc MB0 motif suggested the L24 of N-Myc as the complementary amino acid to the 30th position of TPX2. This observation reflects that N-Myc has two leucines (L24 and L61), protruding to two hydrophobic tunnels of AurA. On the other hand, TPX2 holds two aromatic amino acids (F30 and Y8) for tunnel interactions (Figs. 6 and 7). These findings provide insights into why the N-Myc fragment corresponding to residues 61–89 was not stable in the 5g1x complex. First, the interactions formed by the small aliphatic amino acids such as leucine would be weaker than those formed by the aromatic counterparts in TPX2^34^. Furthermore, the second tunnel that was spotted at the N-terminal lobe of AurA was more confined than the first tunnel, which was found at the lower region of the central lobe. Due to this confinement, the second tunnel at the N-terminal lobe is likely to serve as a better interaction surface for hydrophobic interactions. K250, which is among the first tunnel amino acids, was spotted to form persistent salt bridge interaction with both N-Myc and TPX2, contributing to the intermolecular interaction network around the first tunnel (Fig. 8). K250 of AurA particularly interacted with the E54 of N-Myc. Overall, missing electrostatic interaction of between E54 (Fig. 8) and the hydrophobic interaction of L24 (Fig. 7) in the incomplete N-Myc fragment (61–89) in part addressed its reduced affinity (Table 2).

In relation to stability analysis, we stress that the large disorder within these structures would suggest that enthalpy alone fails to reflect binding free energy of complex. Given the large disorder in the partner structures, the entropy term, which was lacking in the binding free energy ($\Delta\Delta$G) calculations (Table 2), becomes critical as it can substantially affect the binding free energy of the partners. The completed N-Myc (19–89) is composed of $\sim$30% (21/71) hydrophobic and 27% (19/71) charged amino acids. Particularly, the hydrophobic amino acids in this fragment may lead to conformational changes that could also affect the complex stability through entropy rather than enthalpy^40^. This paradigm pertaining to the importance of the entropy for the stability of intrinsically disordered proteins, also means that partial dissociation of the short N-Myc fragment (61–89) that was captured by repeated MD simulations (Fig. 5), would not necessarily reflect a macroscopic behaviour of the complex. Whilst our MD simulations and binding free energy predictions propose that all three N-Myc models constructed in this study are more stable than the N-Myc fragment captured in the crystal structure (61–89), we underline the need of experiments that would conclusively validate these assertions.

Some inhibitors of AurA alter the relative orientation of the N- and C-terminal lobes of AurA, leading to a rearrangement on the surface that the N-Myc fragment (61–89) binds to^19^. This has been proposed as the indirect inhibition mechanism of some kinase inhibitors such as MLN8054 and MLN8237 for formation of the AurA and the N-Myc complex, i.e. a wedge effect of the inhibitor on the orientations of AurA lobes^19^. Our analysis underscored that the N-Myc interaction with the N-terminal lobe of AurA was more stable than with the activation loop of AurA, which was captured in the crystal complex (5g1x). In fact, the N-Myc interaction with the N-terminal lobe of AurA was alone sufficient to stabilize the complex without any other interactions formed with the C-terminal lobe and/or activation loop of AurA. Thus, the AIR or parts of AIR may bind to the N-terminal lobe of AurA without being affected by a change in the orientations of the AurA lobes. Because the AIR of N-Myc, particularly the MB0 motif, can independently form a stable complex with the N-terminal lobe of AurA, a change in the relative orientations of the AurA lobes likely exert an insignificant impact on this interaction. Thus, recognizing that distinct fragments of AIR could form stable complexes with distinct lobes/region of AurA, a wedge effect by some AurA KIs on the relative orientations of AurA lobes may fail to account for a complete inhibition of the interaction between AurA and N-Myc. Overall, insights garnered by this study underscore the promise of direct inhibitors targeting the N-terminal lobe of AurA. The completed AurA and N-Myc complex presented here could facilitate the development of these specific inhibitors.

## Methods

### AurA structures

A total of 159 structures of the Aurora A (AurA) kinase domain were collected from the Protein Data Bank (PDB) as of January 2023 (Supplementary Fig. S1a,b, Supplementary Table S1). To assess structural similarity, principal component analysis (pca) was performed on the C$\alpha$ trace of this PDB ensemble, excluding missing or unmodeled regions using Bio3D^41^. AurA complexes were selected based on their separation on the pc1–pc2 score plots. A total of six distinct structures with the PDB identifiers of 2wtv, 2wtw, 3e5a, 4j8m, 4jbq and 5g1x were selected^19,30,36,42,43^. Missing non-terminal regions were constructed by MODELLER^44^. Particularly, the TPX2 structure in the 3e5a complex and the activation loop of AurA in the 2wtw were modeled prior to simulations. Mutated amino acids and kinase inhibitors/ADP residing at the active site were preserved while other molecules were removed from the structures.

### Molecular dynamics simulations

Molecular dynamics systems were generated by placing the selected AurA complexes in a cubic box of water with a padding distance of 10 Å. Solvated systems were neutralized using Na$^{+}$ and Cl$^{-}$ ions and resulting MD systems were simulated by the NAMD engine (v2.13)^45^ with the CHARMM36 force field including the correction map^46–48^. Water was modeled using the TIP3P model^49^. General CHARMM force field (cGENFF) was used to obtain the parameters of the small molecules^50–52^. Energy minimization was performed using the default algorithm of NAMD for 10,000 steps followed by a multi-step equilibration process as described^38^. First, a short NVT equilibration of 250 ps was applied to only solvent molecules fixing protein atom. Next, another short NVT simulation was applied to only protein atoms fixing the solvent molecules. The last NVT step wherein all atoms moved freely was carried out. Finally, the systems were simulated in an NPT ensemble for 1 ns at constant pressure of 1 atm and temperature of 310 K using the Langevin thermostat and piston pressure method^53–55^. Periodic boundary conditions were applied to all systems. Particle-mesh Ewald summation was used for long-range electrostatic interactions with a grid spacing of 1.2 Å and a cut-off value of 12 Å was used for non-bonded interactions^56^. Production runs were also conducted using NPT ensembles, which lasted for 0.5–1 $\upmu$s at constant pressure of 1 atm and temperature of 310 K using the Langevin thermostat and piston pressure method.

### Data analysis

Visualization of structures and trajectories were carried out by VMD (v1.9.4a) or UCSF Chimera (1.17.3)^57^. 2D-RMSD plots were obtained by MDAnalysis^58,59^. Essential dynamics were extracted using principal component analysis by Bio3D^41^. Tunnel analysis was carried out by the Caver 3.0 tool using a cut-off value of 1.5 Åfor the bottleneck radius^60^. C$\alpha$–C$\alpha$ distances were measured using VMD scripts. VMD (v1.9.4a) movie plugin and VideoMach were used to produce movies.

### MM-GBSA calculations

Binding free energy of the complexes was estimated using AMBER scoring^61^ that uses the MM-GBSA method with AMBER force field^62^. Five MD poses were collected from the last half of each trajectory and and used as inputs. A three-step calculation scheme consisting of pre/post-MD energy minimization and MD steps was applied. A 2000-step minimization was initially performed using the conjugate gradient method. Then, a short MD simulation was applied using NPT ensemble and Langevin dynamics for temperature (310 K) and pressure (1 atm) controls for 5000 steps, followed by the last energy minimization of 2000 steps. All of the protein and solvent atoms were allowed to move freely during these MD calculations. A nonbonded cut-off of 18 Åwas used and $T \Delta S$ term is omitted due to its high computational cost. We optimized the protocol for AMBER scoring on the crystal complex of 3e5a, assessing different calculation schemes as described^63^. The binding free energy ($\Delta G_{binding}$) was estimated as the free energy difference between the energy of the complex and of the individual receptor and ligand structures (Eq. 1).

1 $$\begin{aligned}{} & {} \Delta G_{binding} = \Delta G_{complex} - (\Delta G_{receptor} + \Delta G_{ligand}) \end{aligned}$$

2 $$\begin{aligned}{} & {} \Delta G = \Delta E_{MM} + \Delta G_{solvation} - T \Delta S \end{aligned}$$

The $\Delta G$ term consists of enthalpy ($\Delta H$) and entropy ($T \Delta S$) terms. $\Delta H$ is calculated by MM-GBSA as the sum of the molecular mechanical energy ($\Delta E_{MM}$) and the solvation free energy ($\Delta G_{solvation}$) using the Eq. (2). $\Delta E_{MM}$ is the sum of electrostatic, intramolecular and van der Waals interaction energies. $\Delta G_{solvation}$ contains polar ($\Delta G_{polar}$) and nonpolar ($\Delta G_{nonpolar}$) components. The latter ($\Delta G_{nonpolar}$) is estimated by the modified generalized Born (GB) model developed by Onufriev et al.^64^. $\Delta G_{nonpolar}$ was calculated by the LCPO algorithm based on SASA^65^. The calculated scores of $\Delta G_{binding}$ ultimately represented $\Delta H$ of binding.

### Supplementary Information

Supplementary Information 1.

Supplementary Video S1.

## Data Availability

3D coordinates of the complete AurA and N-Myc model produced by this study were deposited to https://github.com/timucinlab/aura-nmyc.
