# Supplementary material for: Computational completion of the Aurora interaction region of N-Myc in the Aurora a kinase complex
Source: Sci Rep. 2023 Oct 26;13:18399. doi: 10.1038/s41598-023-45272-3 (PMC10603048; doi:10.1038/s41598-023-45272-3)
Supplement: Supplementary file 1 — Supplementary Information 1. [file 41598_2023_45272_MOESM1_ESM.pdf]

Supporting Information
Computational Completion of the Aurora Interaction Region of N-Myc in the Aurora A Kinase Complex

Pinar Altiner[1], Suleyman Selim Cinaroglu[2], Ahmet Can Timucin[3,*], and Emel Timucin[4,*]

[1]Institut de Pharmacologie et de Biologie Structurale, Université Paul Sabatier, CNRS, 205 Rte de Narbonne, 31400 Toulouse, France
[2]Department of Biochemistry, University of Oxford, South Parks Road, Oxford, OX1 3QU, UK
[3]Department of Molecular Biology and Genetics, Faculty of Engineering and Natural Sciences, Acibadem University, Istanbul 34752, Turkey
[4]Department of Biostatistics and Medical Informatics, School of Medicine, Acibadem University, Istanbul 34752, Turkey
[*]Corresponding authors: emel.timucin@acibadem.edu.tr, ahmet.timucin@acibadem.edu.tr

List of Tables

S1 AurA PDB structures . . . 2
S2 MD system details . . . 2

List of Figures

S1 (a) Pairwise sequence similarity (%) and (b) Cα RMSD values were given for 159 AurA KD structures collected from PDB as of January 2023. . . . 3
S2 2D-RMSD heatmaps were calculated for the Cα atoms. . . . 4
S3 Panels show (a) proportion of variance of the first six pcs and (b) score plots of the pc1-pc2. . . 5
S4 Fluctuation analysis of the AurA and its protein partners analyzed in MD simulations. Error bars were show for the top panel displaying the fluctuations of AurA-MBI complex representing standard deviation of fluctuations from four replicate simulations. The AurA/MBI system was simulated five times and the fluctuations were shown as mean±sd, . . . 6
S5 MM-GBSA based binding free energy scores calculated for 10 different MD snapshots. 5g1x (5) system was simulated for 1 μs. . . . 6
S6 Distribution of Cβ-Cβ distances between the first tunnel amino acids and the closest amino acid of L61 corresponding to MBI motif in the N-Myc fragment (61-89) of 5g1x complex. Simulation number (5) was lasted for 1μs. . . . 7
S7 (a) Panel shows ribbon representations of AurA and NMyc (61-89) fragment after multi-step equilibration (eq.) and production (prod.) runs. N-Myc(61-89) fragment: orange, AurA activation loop: green). (b) Reduced trajectory of AurA complexes were shown in ribbon representation (red: start, white: middle, blue: end). AurA N- and C-terminal lobes are colored silver and tan, respectively. Activation loop is colored green. Bottom panel shows a rotated view of top representations. Structures were visualized by VMD (v1.9.4). . . . 8

Tables

Table S1 AurA PDB structures

2WTV, 2WTW, 3E5A, 4J8M, 4JBQ, 5G1X, 1MQ4, 1MUO, 1OL5, 1OL6, 1OL7, 2BMC, 2C6D, 2C6E, 2DWB, 2J4Z, 2J50, 2NP8, 2W1C, 2W1D, 2W1E, 2W1F, 2W1G, 2WQE, 2X6D, 2X6E, 2X81, 2XNE, 2XNG, 2XRU, 3COH, 3EFW, 3FDN, 3H0Y, 3H0Z, 3H10, 3HA6, 3K5U, 3LAU, 3M11, 3MYG, 3NRM, 3O50, 3O51, 3P9J, 3QBN, 3R21, 3R22, 3UNZ, 3UO4, 3UO5, 3UO6, 3UOD, 3UOH, 3UOJ, 3UOK, 3UOL, 3UP2, 3UP7, 3VAP, 3W10, 3W16, 3W18, 3W2C, 4B0G, 4BN1, 4BYI, 4BYJ, 4C3P, 4C3R, 4CEG, 4DEA, 4DEB, 4DED, 4DEE, 4DHF, 4J8N, 4JAI, 4JAJ, 4JBO, 4JBP, 4O0S, 4O0U, 4O0W, 4PRJ, 4UYN, 4UZD, 4UZH, 4ZS0, 4ZTQ, 4ZTR, 4ZTS, 5AAD, 5AAE, 5AAF, 5AAG, 5DN3, 5DNR, 5DOS, 5DPV, 5DR2, 5DR6, 5DR9, 5DRD, 5DT0, 5DT3, 5DT4, 5EW9, 5G15, 5L8J, 5L8K, 5L8L, 5LXM, 5OBJ, 5OBR, 5ODT, 5ONE, 5ORL, 5ORN, 5ORO, 5ORP, 5ORR, 5ORS, 5ORT, 5ORV, 5ORW, 5ORX, 5ORY, 5ORZ, 5OS0, 5OS1, 5OS2, 5OS3, 5OS4, 5OS5, 5OS6, 5OSD, 5OSE, 5OSF, 5ZAN, 6C2R, 6C2T, 6C83, 6CPE, 6CPF, 6CPG, 6GRA, 6HJJ, 6HJK, 6I2U, 6R49, 6R4A, 6R4B, 6R4C, 6R4D, 6Z4Y, 7AYH, 7AYI, 7O2V

Table S2 MD system details

Complex	Total	Protein	Water	x (Å)	y (Å)	z (Å)
5G1X:61-89 (rep1)	38567	4797	33729	74.5	74.8	75.4
5G1X:61-89 (rep2)	30350	4797	25506	74.3	71.7	62.1
5G1X:61-89(rep3)	37693	4797	32787	74.3	78.7	70.1
5G1X:61-89 (rep4)	42701	4797	37857	82.3	79.7	70.1
5G1X:MB0	32462	5003	27414	80.7	64.9	68.1
5G1X:MB0/MBI	38404	5439	32910	74.6	74.8	75.4
5G1X:TPX2/MBI	62514	5345	57120	86.7	86.7	86.6
3E5A:TPX2	60248	4926	55257	85.6	85.7	85.7
4JBQ	29983	4373	25545	75.5	70.4	61.8
4J8M	36662	4385	32208	72.8	72.8	72.8
2WTW	62686	4336	58299	87.8	87.8	87.7
2WTV	44775	4382	40344	78.3	78.6	78.5
2WTV (no Phos)	36579	4377	32148	73.7	73.8	73.6

Number of atoms and system size details were given.

Figures

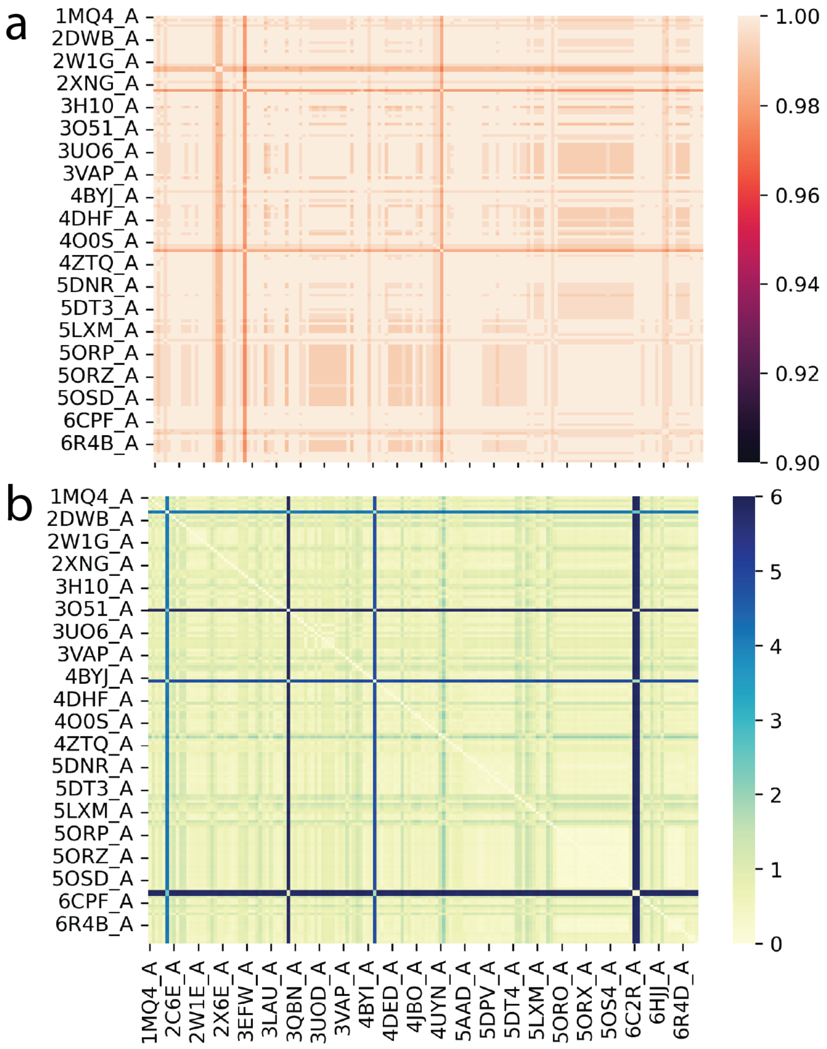

Figure S1 (a) Pairwise sequence similarity (%) and (b) Cα RMSD values were given for 159 AurA KD structures collected from PDB as of January 2023.

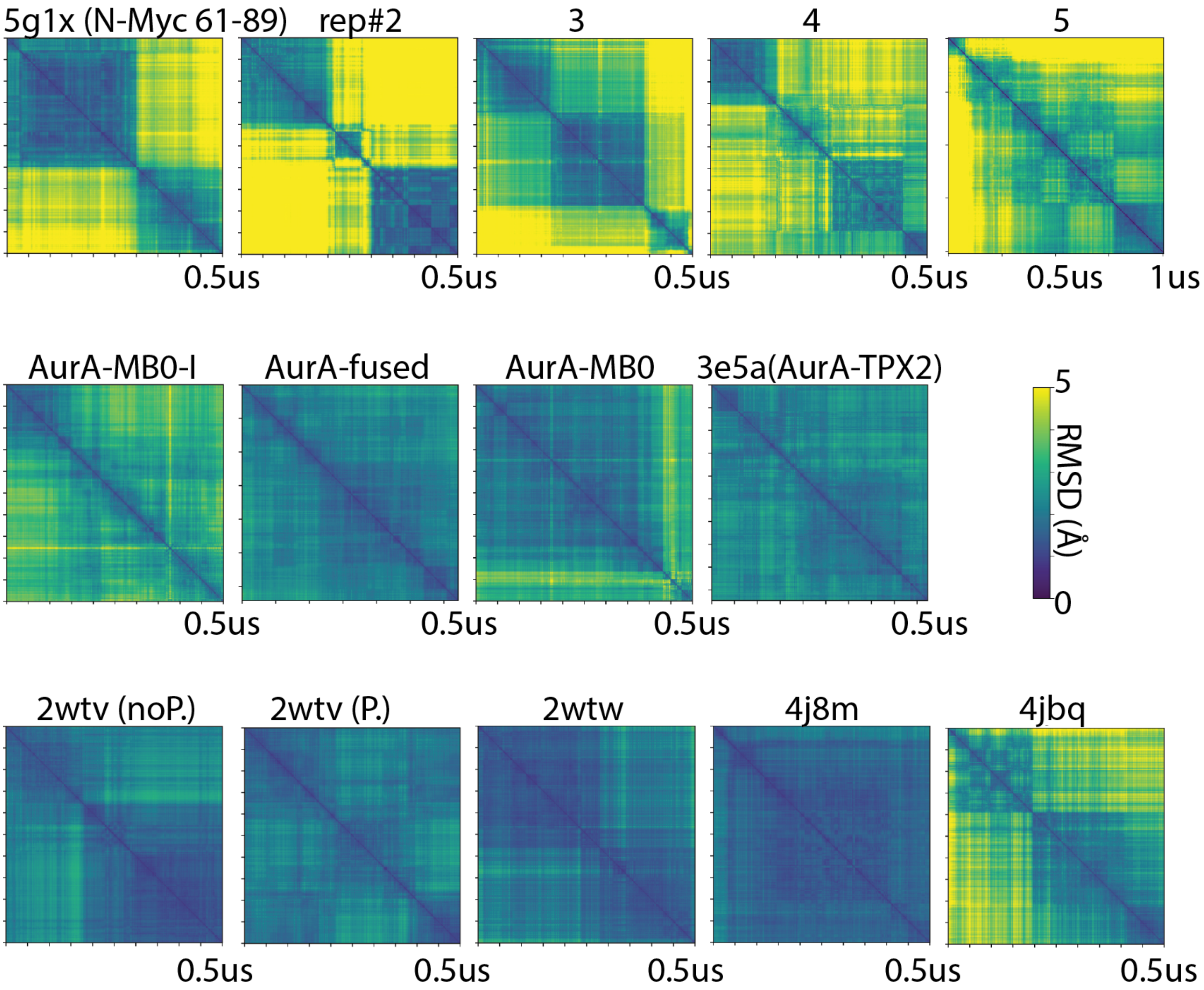

Figure S2 2D-RMSD heatmaps were calculated for the Cα atoms.

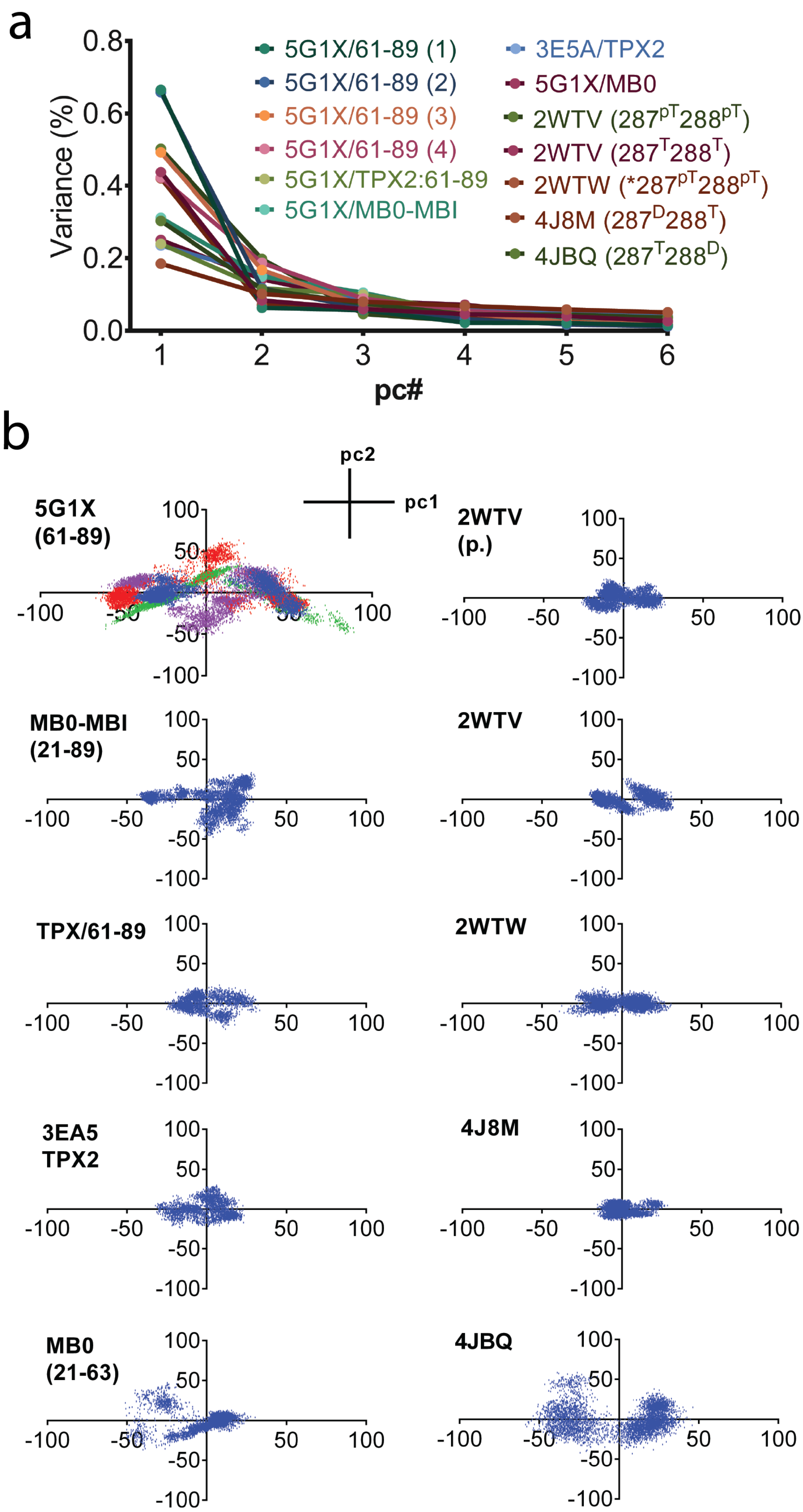

Figure S3 Panels show (a) proportion of variance of the first six pcs and (b) score plots of the pc1-pc2.

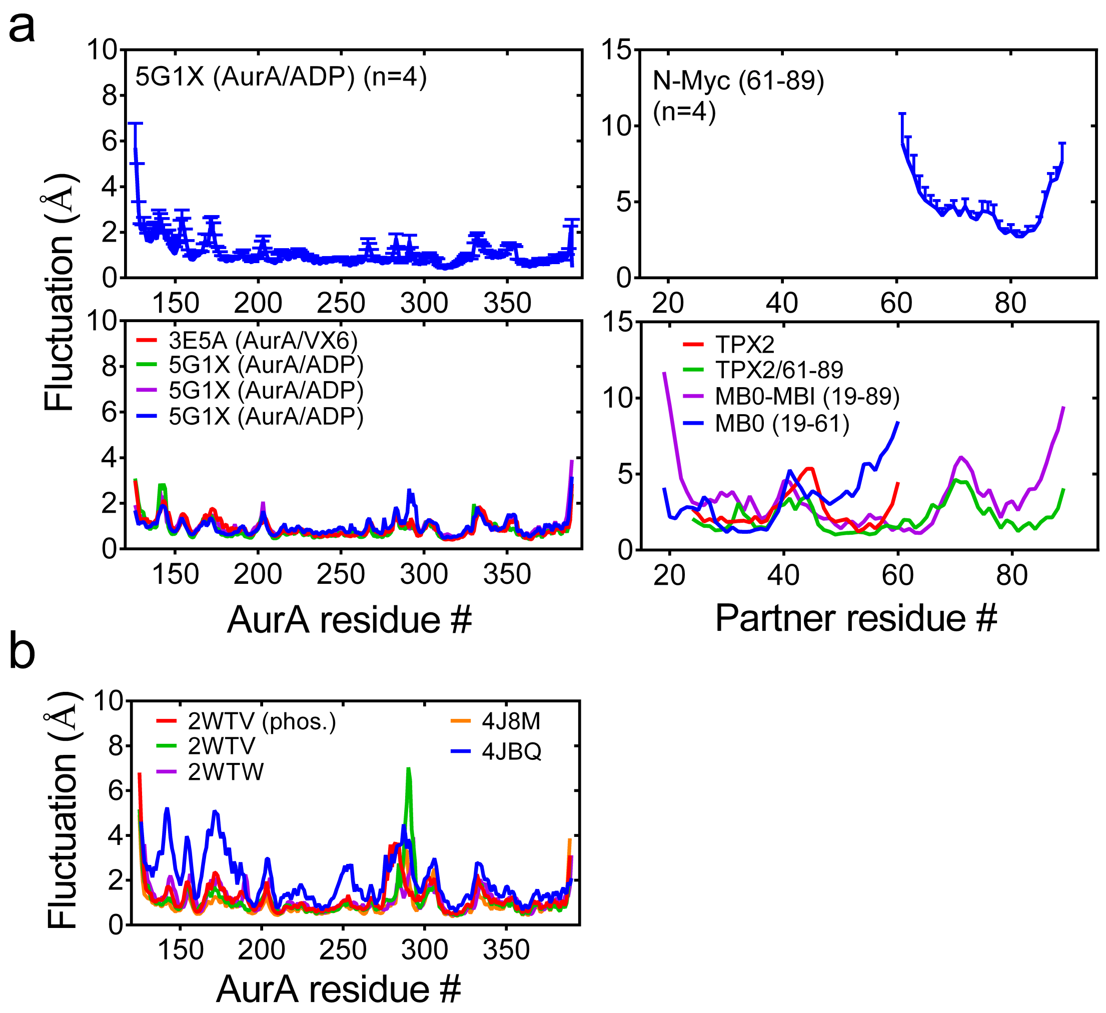

Figure S4 Fluctuation analysis of the AurA and its protein partners analyzed in MD simulations. Error bars were show for the top panel displaying the fluctuations of AurA-MBI complex representing standard deviation of fluctuations from four replicate simulations. The AurA/MBI system was simulated five times and the fluctuations were shown as mean±sd,

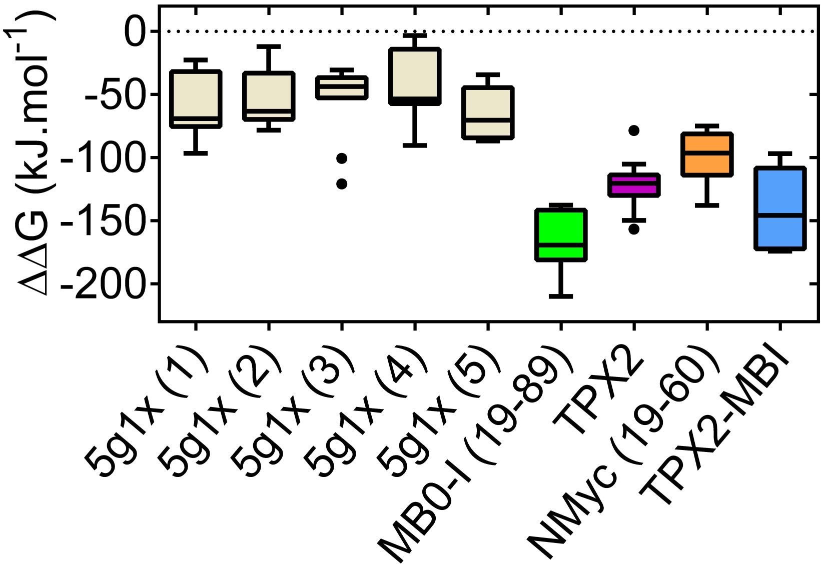

Figure S5 MM-GBSA based binding free energy scores calculated for 10 different MD snapshots. 5g1x (5) system was simulated for 1 μs.

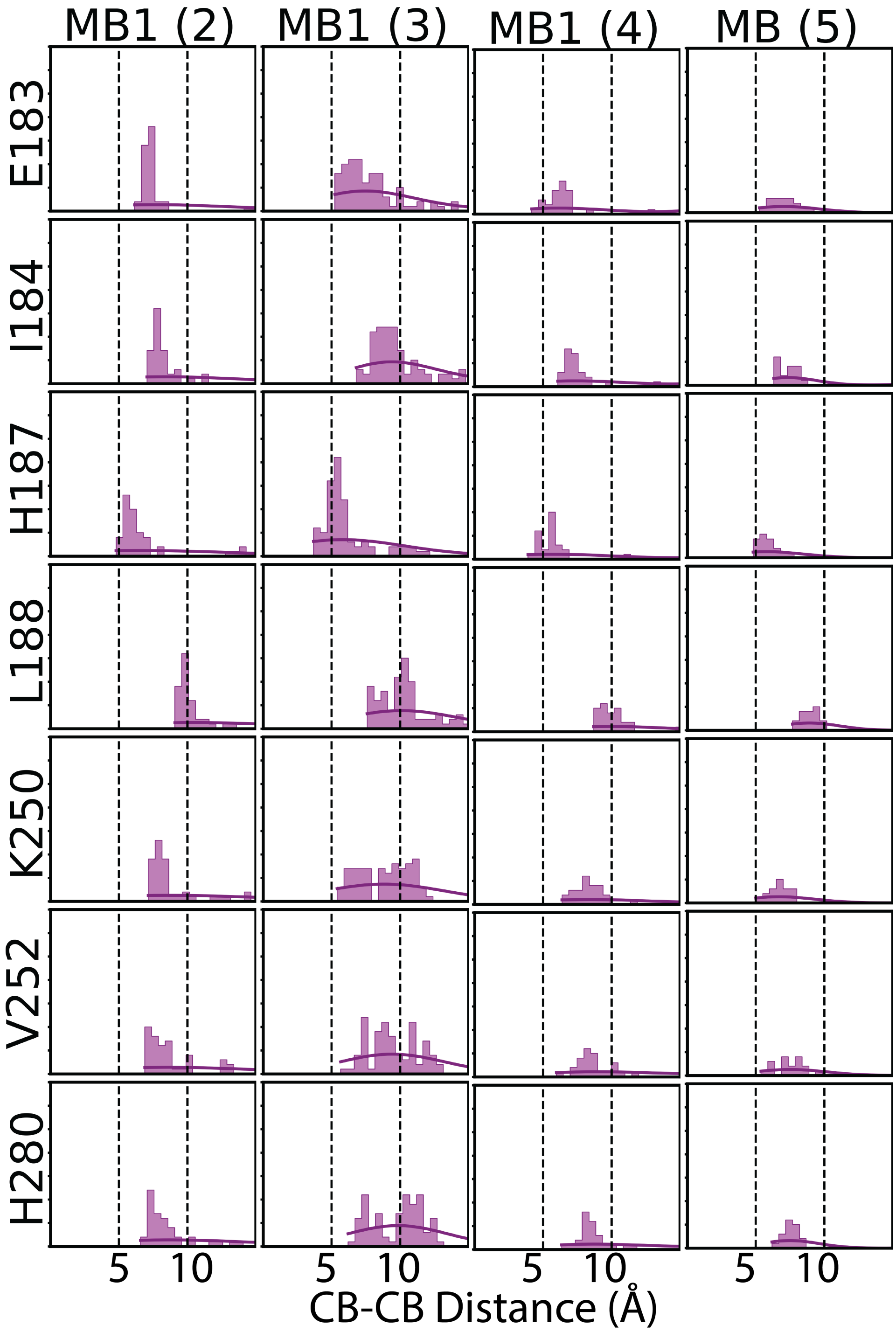

Figure S6 Distribution of Cβ-Cβ distances between the first tunnel amino acids and the closest amino acid of L61 corresponding to MBI motif in the N-Myc fragment (61-89) of 5g1x complex. Simulation number (5) was lasted for 1μs.

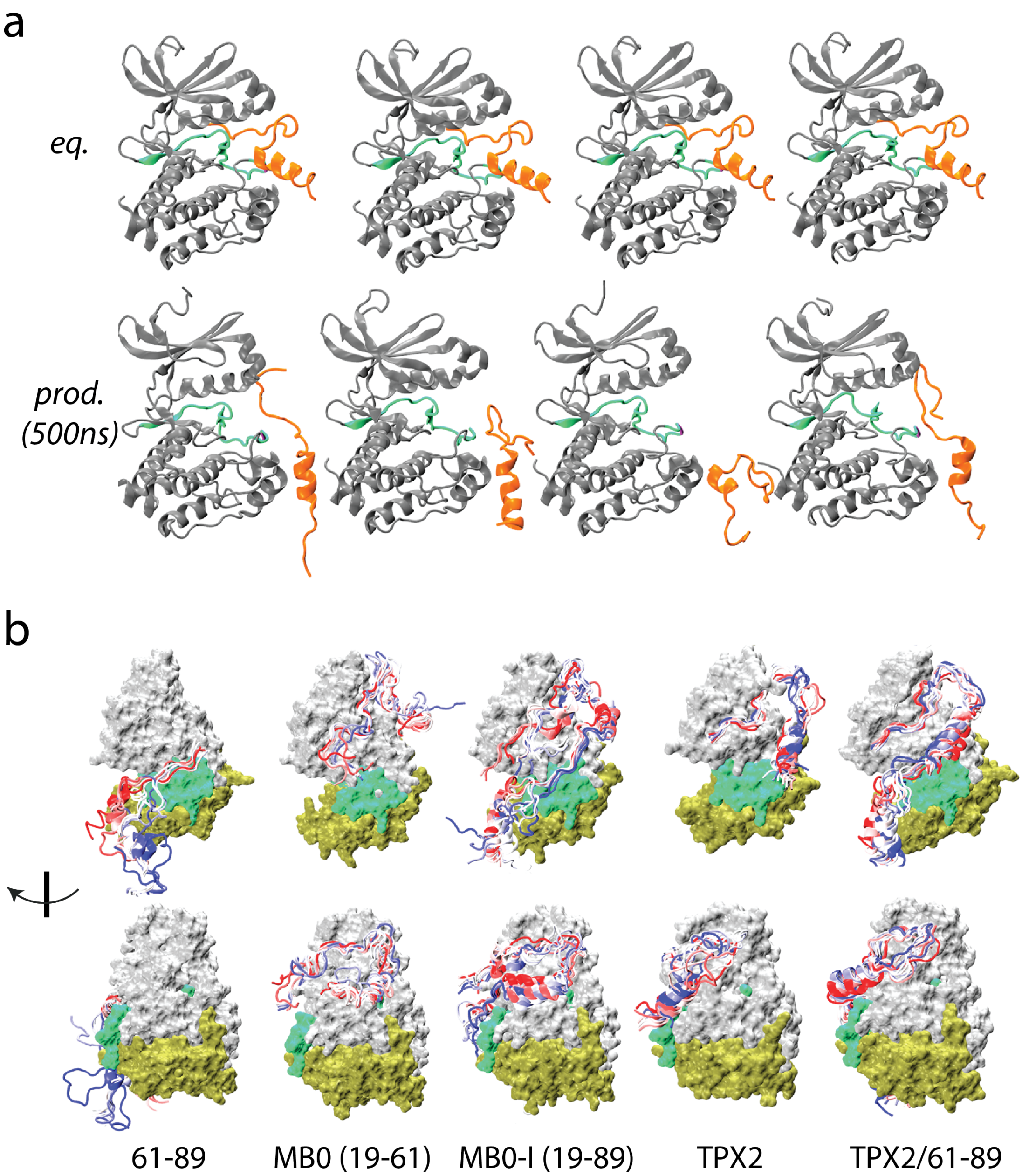

Figure S7 (a) Panel shows ribbon representations of AurA and NMyc (61-89) fragment after multi-step equilibration (eq.) and production (prod.) runs. N-Myc(61-89) fragment: orange, AurA activation loop: green). (b) Reduced trajectory of AurA complexes were shown in ribbon representation (red: start, white: middle, blue: end). AurA N- and C-terminal lobes are colored silver and tan, respectively. Activation loop is colored green. Bottom panel shows a rotated view of top representations. Structures were visualized by VMD (v1.9.4).
